# Throughput and compartmentalization shape bacterial recovery in droplet- and plate-based cultivation of hydrogen-oxidizing communities

**DOI:** 10.1093/ismeco/ycag289

**Published:** 2026-10-09

**Authors:** Wannes Nauwynck, Astrid Rombouts, Karoline Faust, Nico Boon

**Affiliations:** Center for Microbial Ecology and Technology (CMET), Department of Biotechnology, Ghent University, Frieda Saeysstraat 1, 9052 Gent, Belgium; Laboratory of Molecular Bacteriology (Rega Institute), Department of Microbiology, Immunology and Transplantation, KU Leuven, Herestraat 49, 3000 Leuven, Belgium; Centre for Advanced Process Technology for Urban Resource Recovery (CAPTURE), Frieda Saeysstraat 1, 9052 Ghent, Belgium; Center for Microbial Ecology and Technology (CMET), Department of Biotechnology, Ghent University, Frieda Saeysstraat 1, 9052 Gent, Belgium; Centre for Advanced Process Technology for Urban Resource Recovery (CAPTURE), Frieda Saeysstraat 1, 9052 Ghent, Belgium; Laboratory of Molecular Bacteriology (Rega Institute), Department of Microbiology, Immunology and Transplantation, KU Leuven, Herestraat 49, 3000 Leuven, Belgium; Center for Microbial Ecology and Technology (CMET), Department of Biotechnology, Ghent University, Frieda Saeysstraat 1, 9052 Gent, Belgium; Centre for Advanced Process Technology for Urban Resource Recovery (CAPTURE), Frieda Saeysstraat 1, 9052 Ghent, Belgium

**Keywords:** cultivation methods, microbial community, droplet microfluidics, hydrogen-oxidizing bacteria, hydrogen-oxidizing communities

## Abstract

Isolation methods are essential for recovering microbial diversity for physiological characterization. Droplet-based cultivation frequently shows greater taxonomic recovery than conventional plating, but the basis of this advantage remains unclear. Here, we used hydrogen-oxidizing enrichment communities as a tractable model system to compare droplet- and plate-based cultivation and explicitly separate throughput from cultivation-format effects. Both methods combined achieved high recovery (~90%), with droplets recovering more Operational Taxonomic Units (OTUs), particularly under heterotrophic conditions (77.8% vs. 51.7%). However, much of this advantage was explained by the substantially higher number of cells screened in droplets. Comparison with previous droplet-cultivation studies showed that differences in founder-cell numbers are also common in the literature and are often not separated from cultivation-format effects, potentially inflating the apparent biological advantage of droplets. After throughput normalization, a significant residual droplet advantage remained under heterotrophic conditions (100% vs. 90.0%), whereas no clear difference remained under autotrophic conditions. Droplet growth showed compartmentalization of nutrients, OTU growth tended to be more even in droplets, and the residual recovery advantage was concentrated among lower-abundance taxa, patterns consistent with reduced competitive dominance but not sufficient to establish competition as the causal mechanism. Overall, our results pick apart increased recovery in droplet cultivation as a combination of higher throughput and cultivation-format-specific effects.

## Introduction

The foundations of microbiology were laid in the 19th century when Robert Koch developed plate-based cultivation methods that enabled the isolation of pure cultures. While plate culturing revolutionized our understanding of microbial life, it captured only a small fraction of microbial diversity [1, 2]. Late 20th-century molecular advances revealed the extent of this limitation, showing that most microorganisms remain uncultured [3–5]. The great plate count anomaly still rings true today, with a recent metagenomic census estimating that only around 10% of sequenced bacterial taxa have a cultivated representative [6]. Without representative axenic isolates, the vast diversity in bacterial physiology, ecological roles, and potential applications of uncultivated bacteria remains unexplored.

A myriad of factors is thought to underlie bacterial uncultivability on plates: unknown nutritional requirements, overgrowth by fast-growing taxa, failure to resuscitate dormant phenotypes, removal of obligate microbial partners, failure to capture rare taxa, and failure to recreate spatial structure or physicochemical environmental conditions [7–10]. Targeted methods have been developed to address limitations of plate cultivation: diffusion chambers and the iChip enable *in situ* growth [11, 12], cell sorting is used to sort active cells with desired taxonomy [13] or functionality [14], dilution-to-extinction cultivation minimizes competition by physically separating individual cells into isolated growth vessels [15, 16]. Low-abundance taxa have been isolated using antibody-based cell sorting informed by reverse genomics [17].

A more recent development to tackle plate-linked limitations is the increasing use of water-in-oil emulsions to encapsulate single cells in a small droplet of medium to cultivate cells [18–23]. Droplet-based cultivation techniques make use of picoliter to nanoliter water-in-oil emulsions (droplets). They can be made in ultrahigh (100–10.000 per second) throughput, allowing individual cells of entire complex microbial communities to be encapsulated in separate droplets [24]. Zengler *et al.* (2002) were the first to report the use of microcompartments to successfully cultivate microorganisms, showing a broad taxonomic recovery and cultivation of previously uncultivated clades of marine bacteria from a sample of the Sargasso Sea [25]. Recent reports have followed this seminal paper, all reporting an increase in recovered taxonomic richness when cultivating bacteria from environmental communities, comparing droplet cultivation to conventional plating [18–21, 23]. From these reports, it is unclear, however, what the dominant mechanism is behind this increased recovery of richness of droplets compared to plates. Droplets differ from plates in several ways: they do not require the organism to switch growth states (planktonic to surface-attached), they are more closed in nature, possibly limiting interactions between growing populations, and they provide a distinct microenvironment within the droplet [26]. In addition, droplet systems boast an intrinsically greater sampling depth than conventional plating, increasing the probability of capturing low-abundance taxa. Because this throughput advantage is not always explicitly separated from other method-associated effects [18, 23], increased recovery in droplets may reflect greater sampling depth aside from properties of the cultivation format itself.

Here, we aimed to explicitly look at the contribution of increased throughput and to assess whether differences between droplets and plates remained after accounting for this effect and what the likely causes are for these differences. To do this, we used hydrogen-oxidizing enrichments as a tractable model community in which major causes of uncultivability, including unknown physicochemical requirements, dormancy, and failure to recreate spatial structure, were reduced, but other potential causes like disrupting of obligate mutualistic interactions or introduction of competitive interactions (during isolation) could still occur. Rather than completing the full isolation workflow to obtain axenic strains, we used successful growth after separation (plate spreading or droplet encapsulation) as a proxy for isolation success. We hypothesized that stronger physical separation in droplets reduces interspecies competition and thereby supports detectable growth of a broader range of taxa compared to plating.

## Materials and methods

### Enrichment communities and cultivation media

Three hydrogen-oxidizing enrichment communities were established from a methylotrophic reactor, a hydrogenated trickling filter reactor [27] and post-aeration sludge from a water treatment plant (RWZI, Gent, Belgium). Communities were enriched for four weeks in mineral -Deutsche Sammlung von Mikroorganismen und Zellkulturen (DSMZ)-81 medium under a 73% H_2_, 18% CO_2_, and 9% O_2_. Atmosphere at 28°C, with 1:10 transfers performed twice weekly. Full medium composition and enrichment procedures are provided in the Supplementary Methods.

Autotrophic cultivation used mineral DSMZ-81 medium. Heterotrophic cultivation used the same basal medium supplemented with 0.2% (w/v) yeast extract, 0.6% (w/v) pyruvate and 0.6% (w/v) glucose. Solid media contained 10 g/l gellan gum.

### Inoculum preparation

Before cultivation experiments, enrichment cultures were transferred 1:10 into fresh mineral DSMZ-81 medium and grown overnight under autotrophic conditions. Cultures were harvested during exponential growth, washed three times in ice-cold DSMZ-81 medium, and resuspended to generate a concentrated inoculum stock. The same stock was subsequently diluted into the appropriate autotrophic or heterotrophic medium for plate- and droplet-based cultivation.

### Droplet and plate cultivation

For droplet cultivation, inocula were adjusted to 2 × 10^6^ cells/ml with heterotrophic conditions being diluted with rich DSMZ-81 and autotrophic conditions being diluted with mineral DSMZ-81; for 20 pl droplets this corresponds to a Poisson parameter of λ = 0.04, yielding 3.8% single-cell occupancy (P_Poisson_(X = 1) = (0.04^1^*e^-0.04^)/(1!) = 3.8%), with the remainder of the droplet predominantly empty. Droplets consisted of HFE7500 containing 0.5 w/w% Pico-Surf and were incubated for 10 days at 28°C and 120 rpm. Detailed device fabrication and droplet-generation parameters are provided in the Supplementary Methods. Following incubation, droplets were coalesced for flow-cytometric cell counting and DNA extraction.

For plate cultivation, 100 ul of serially diluted inocula were spread on mineral or rich DSMZ-81 gellan-gum plates for autotrophic and heterotrophic cultivation, respectively. Autotrophic plates were incubated under 73% H_2_, 18% CO_2_, 9% O_2_, and all plates were incubated at 28°C for 8 days. Entire plate surfaces were scraped into PBS for flow cytometry and DNA extraction. Experiments were performed in biological duplicate for each enrichment.

### Growth validation and cell quantification

Growth was independently validated by microscopy and SYBR Green staining. Coalesced droplet samples and scraped plate biomass were stained with 1× SYBR Green I and analyzed using an Accuri C6+ flow cytometer to determine total cell concentrations. Additional staining of intact double emulsions was used to confirm DNA-containing biomass within droplets; detailed procedures are provided in the Supplementary Methods.

### DNA extraction, library preparation and sequencing

DNA was extracted using the DNeasy PowerSoil Pro kit and full-length 16S rRNA amplicons were sequenced using PacBio Kinnex chemistry on a Revio platform. Reads were processed using the PacBio HiFi-16S workflow, ASVs were inferred using DADA2 and classified against SILVA 138.1. Full library-preparation and bioinformatic parameters are provided in the Supplementary Methods.

### Absolute abundance, fold change, and OTU growth

Sequencing libraries were rarefied within enrichment, and ASV abundances were corrected for estimated 16S rRNA gene copy number using rrnDB. Copy-number-corrected relative abundances were converted to absolute abundances by multiplying each ASV’s relative abundance by the corresponding total cell concentration measured by flow cytometry. Fold change for each ASV was calculated as:


$${Fold\ Change}_i=\frac{absolute\ abundance\ of\ {ASV}_i\ at\ {t}_{end}}{absolute\ abundance\ of\ {ASV}_i\ at\ {t}_0}$$

ASVs with fold change >2 were classified as showing growth. To reduce the influence of fine-scale sequencing variation, ASVs were clustered within genera at 99.75% 16S rRNA sequence identity; a threshold selected using mock-community validation. Operational taxonomic unit (OTU) growth was represented by the growth status of its most abundant ASV. Detailed rarefaction, copy-number correction, and clustering procedures are provided in the Supplementary Methods.

### OTU growth-evenness analysis

To assess whether population expansion differed between cultivation formats, the analysis was restricted to OTUs showing growth in both methods. Within each enrichment, biological replicate, and cultivation condition, OTU fold changes were median-normalized, and their variability quantified using the interquartile range (IQR). Lower IQR values indicate more similar fold changes among OTUs and therefore more even population expansion. Paired droplet and plate IQR values were compared separately under autotrophic and heterotrophic conditions using paired Wilcoxon signed-rank tests, with Bonferroni correction across the two comparisons.

### Assessment of nutrient containment in droplets

To assess whether growth-limiting nutrients were retained within droplets, cell yield per occupied droplet was compared between low-occupancy (~3.9%) and high-occupancy (~86.5%) autotrophic cultures (2e6/ml and 1e8/ml for 20 pl droplets). Following cultivation, droplets were coalesced and cells quantified by SYBR Green flow cytometry. Droplet numbers were estimated from the recovered emulsion volume. Complete redistribution of a limiting nutrient would theoretically provide ~22-fold greater nutrient availability per occupied droplet under low occupancy.

### Statistical analysis of droplet and plate recovery

Analyses were performed in R 4.5.1. Droplet and plate recovery were compared using paired one-sided bootstrap tests over matched enrichment × biological-replicate pairs (2000 resamples). Multiple comparisons were adjusted as indicated for individual analyses. Detailed statistical procedures and software packages are provided in the Supplementary Methods.

## Results

### Enrichment communities all contain hydrogen-oxidizing core taxa with minor heterotrophic members

To assess cultivability of a set of bacteria, we enriched microbial communities by incubating diverse inocula in a defined mineral medium under a controlled gas atmosphere of H_2_, O_2_, and CO_2_. This approach was chosen based on the expectation that the resulting communities would comprise autotrophic and heterotrophic bacteria, allowing for a clear cultivation strategy of these two groups [28–30] in autotrophic and heterotrophic conditions (Fig. 1a). To ensure broad taxonomic diversity, inocula were sourced from 3 different environments with putative aerobic hydrogenotrophic activity: (i) post-aeration sludge from a municipal wastewater treatment plant, (ii) biomass from a methylotrophic bioreactor, and (iii) a hydrogen-fed reactor used for tertiary treatment of wastewater effluent.

**Figure 1:**
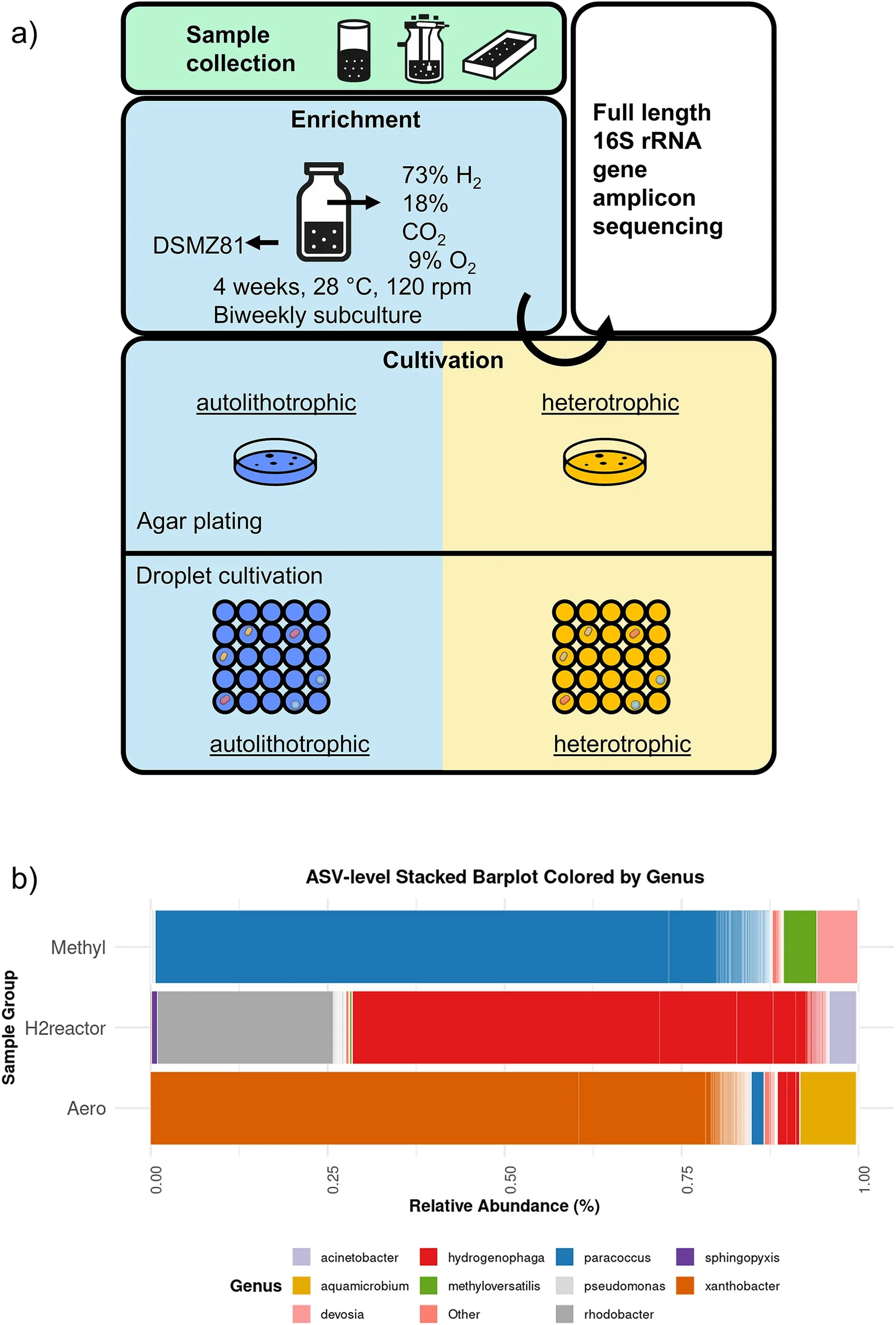
Schematic overview of the experimental approach. Samples were collected from three environments: (i) a hydrogen-fed reactor used for tertiary treatment of wastewater effluent (hydrogen reactor), (ii) a methylotrophic reactor, and (iii) aerobic sludge from a municipal wastewater treatment plant. These samples were subcultured twice weekly in a mineral medium with hydrogen and oxygen as energy sources and carbon dioxide as the sole carbon source. Cultivation was performed using both agar plating and droplet microfluidics under autotrophic and heterotrophic conditions. Full-length 16S rRNA gene sequencing was used to identify the taxa that grew in each condition. b) Relative 16S rRNA gene abundance profiles of the aerobic sludge, methylotrophic reactor, and hydrogen reactor enrichments used for cultivation (grown under hydrogen, oxygen, and CO2 conditions). Average relative abundance shown over two biological replicates. ASVs per genus are indicated with white lines.

Full-length 16S rRNA gene sequencing revealed distinct microbial community compositions across the three enrichments (Fig. 1b). The enrichments showed similar levels of genus richness: the aerobic sludge, methylotrophic reactor, and hydrogen reactor respectively exhibited 33, 30, and 23 distinct bacterial genera.

All enrichments were dominated by one or two genera. Dominant genera were unique for each enrichment (Fig. 1b). The methylotrophic enrichment was dominated by *Paracoccus* (87.7 ± 6.7%), the aerobic sludge enrichment was dominated by *Xanthobacter* (84.5 ± 1.21%) and the hydrogen reactor contained two major genera: *Hydrogenophaga* (67.4 ± 1.6%) and *Rhodobacter* (25.6 ± 1.6%).

Each genus showed distinct amplicon sequence variant (ASV)-level richness across the enrichment conditions. The most abundant genera were also among the most rich. In the aerobic sludge enrichment, *Xanthobacter* exhibited the highest ASV richness with 92 distinct ASVs. The methylotrophic reactor enrichment, *Paracoccus* had the greatest overall within-genus richness, with 113 ASVs detected. In the H_2_ reactor enrichment, several genera contributed high ASV richness, including *Hydrogenophaga* with 74 ASVs, *Acinetobacter* with 65 ASVs, and *Rhodobacter* with 36 ASVs.

In each enrichment condition, several non-dominant genera contributed to the microbial community structure (Fig. 1b). In the aerobic sludge enrichment, these included *Aquamicrobium* (8.2 ± 0.9%), *Hydrogenophaga* (3.2 ± 0.4%), *Paracoccus* (1.9 ± 0.1%), *Rhizobium* (0.80 ± 0.02%), and *Ochrobactrum* (0.3 ± 0.03%).

In the methylotrophic reactor enrichment, prominent but non-dominant genera included *Devosia* (5.7 ± 0.2%), *Methyloversatilis* (4.6 ± 0.7%), *Shinella* (0.70 ± 0.03%), *Pseudomonas* (0.41 ± 0.03%), *Hydrotalea* (0.21 ± 0.02%), *Ferrovibrio* (0.15 ± 0.02%), and *Azospirillum* (0.09 ± 0.004%).

In the hydrogen reactor enrichment, *Acinetobacter* was the most prominent among the non-dominant genera (4.14 ± 0.60%), followed by *Sphingopyxis* (0.87 ± 0.33%), *Pseudomonas* (0.53 ± 0.02%), *Lacunisphaera* (0.29 ± 0.13%), *Methyloversatilis* (0.28 ± 0.02%), *Xanthobacter* (0.13 ± 0.01%), *Paracoccus* (0.13 ± 0.03%), and *Rhizobium* (0.10 ± 0.05%).

These results, together with our data showing consistent gas consumption down to sub-atmospheric pressures (Supplementary Information (SI): Fig. S1) demonstrate that our enrichments were actively consuming their gas atmosphere and effectively selected for taxonomically diverse hydrogen-oxidizing microbial communities, each exhibiting inoculum-specific dominance patterns.

### Droplet- and plate-based cultivation achieve high enrichment cultivation success

To obtain an overall idea of the isolation performance of each method, we determined which OTUs were successfully cultured by either droplet- or plate-based methods and which remained uncultured. Both methods physically separated cells during inoculation, enabling independent growth and clonal expansion from single cells. Although this does not represent a full isolation workflow (i.e. growth followed by purification to axenic cultures), it reflects the ability of a microorganism to grow independently. We therefore use OTU growth as a proxy for isolation success at the OTU level. In droplets, compartmentalization enabled physical separation. On plates, we used a low seeding density (0.1–1 cells/mm^2^) to minimize interactions during colony establishment. As a result, we consider growth to reflect the ability of individual cells to proliferate independently. To calculate cultivation success, we looked at which OTUs grew in at least one bioreplicate for a given method and divided this by how many OTUs were present in the original enrichment.

Growth was confirmed in both cultivation formats using microscopy, SYBR Green staining, and flow cytometry. On plates, visible colony formation was observed after incubation (SI: Fig. S2 and S3), and SYBR Green staining and flow-cytometric analysis of the scraped biomass confirmed the presence of cells. In droplets, microscopy showed cellular growth during incubation (SI: Figs S4–S8). Cellular growth was independently confirmed in two ways: first, intact droplets of autotrophic conditions were converted to double emulsions, stained with SYBR Green, and analyzed by flow cytometry to confirm that DNA-containing cells were indeed present within the droplets (SI: Fig. S9); second, droplets were coalesced after incubation, and the released cells were directly quantified by SYBR Green staining and flow cytometry. Together, these measurements confirmed that the material observed microscopically within droplets represented cells.

To classify growth/non-growth of OTUs, ASVs were clustered within each enrichment at 99.75% 16S rRNA gene sequence identity based on mock-community analysis, which showed that this threshold grouped most error-derived variants while retaining most of the true diversity (see SI for detailed analysis of ASV-level diversity). Growth was determined from fold changes in absolute abundance between the start (t_0_) and end (t_end_) of incubation. Absolute ASV abundance was estimated by multiplying total cell counts by the corresponding 16S rRNA gene relative abundance as described previously [31] and fold change was calculated as abundance at t_end_ divided by abundance at t_0_. An ASV was considered to have grown when its estimated absolute abundance increased by more than twofold over the incubation period. OTU growth was defined by the growth status of its most abundant ASV. Because many within-OTU ASVs occurred at very low abundance (see SI), their fold-change estimates were more susceptible to technical variation, making the dominant ASV a more robust proxy for OTU-level growth. Finally, to calculate cultivation success (= cultivation recovery), we use the amount of OTUs classified as grown and divide it by the total number of OTUs in the inoculum. For autotrophic cultivation, the hydrogen-reactor enrichment was excluded because very limited growth was observed in droplets, likely due to insufficient agitation during incubation.

Cultivation success was high when pooling results for both methods, with 87.9% of OTUs cultivated under autotrophic conditions and 90.2% under heterotrophic conditions (Fig. 2a and b). Only 7 OTUs for autotrophic conditions and 10 OTUs for heterotrophic conditions remained uncultivated. These consisted of 4 distinct *Azospirillum* OTUs, 1 *Bacteroides* OTU, and 2 *Sphaerochaeta* OTUs for autotrophic conditions. For heterotrophic conditions, these were 3 *Bacteroides* OTUs, 1 *Desulfovibrio* OTU, 1 *Hydrotalea* OTU, 1 *Lacunisphaera*, 1 *Pseudoxanthomonas*, 1 *Sphaerochatea*, and 1 *Thiobacillus* OTU. In autotrophic conditions, plate cultivation uniquely recovered 2 OTUs, compared to 8 unique to droplet cultivation. In heterotrophic conditions, plates uniquely recovered 4 OTUs, while droplets recovered 29 exclusively. The total richness of each enrichment varied: 30, 44, and 28 OTUs were detected in the aerobic sludge enrichment, the hydrogen reactor enrichment, and the methylotrophic reactor enrichment, respectively.

**Figure 2:**
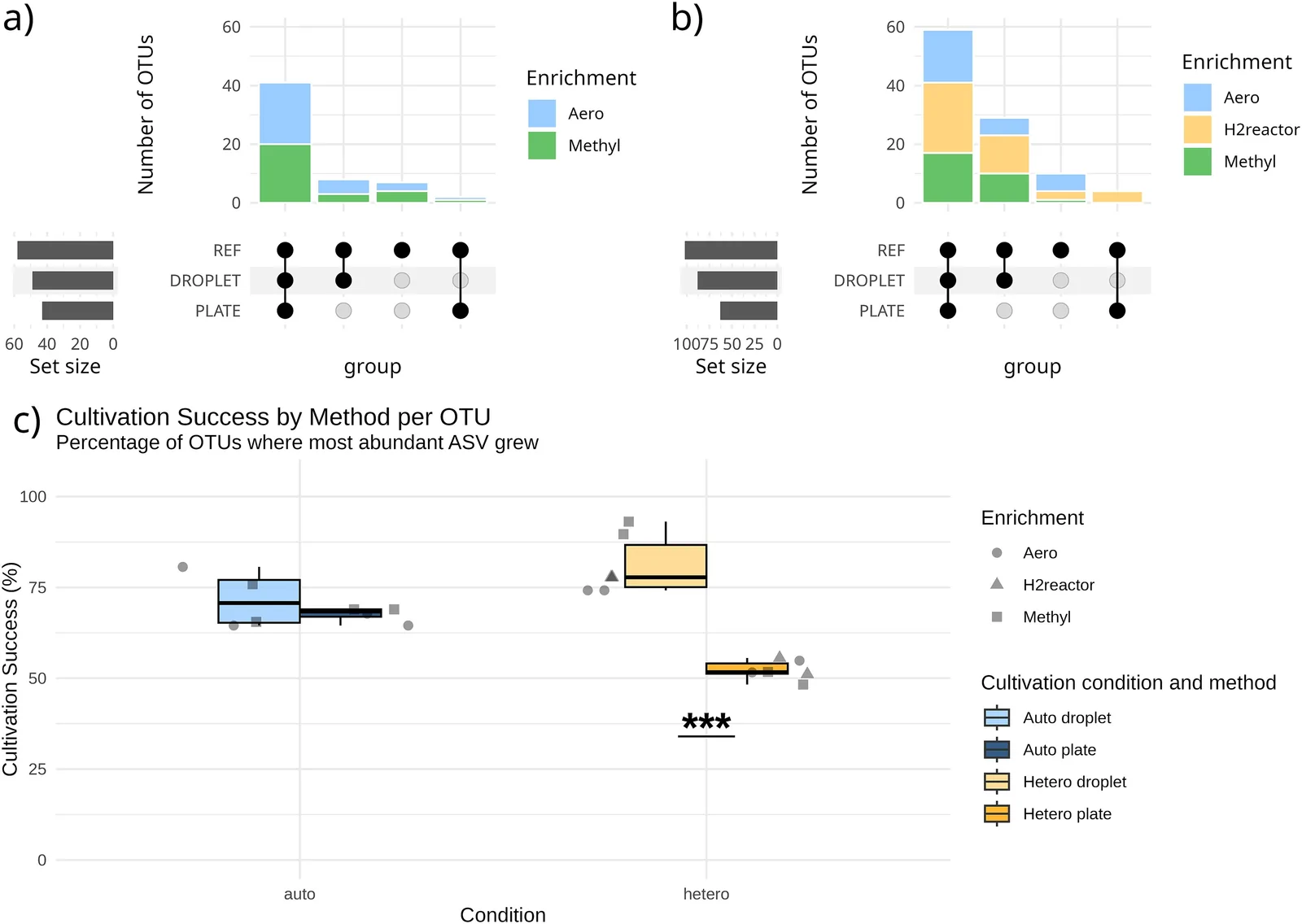
a) Shared and unique OTUs cultivated under each cultivation method in autotrophic (a) and heterotrophic (b) conditions. Bars represent the number of OTUs recovered in each combination of the reference inoculum (REF), DROPLET-based cultivation (DROPLET), and PLATE-based cultivation (PLATE). Colors within the bars indicate the enrichment type from which the OTUs originated (aero: Aerobic sludge, H2reactor: Hydrogen reactor, methyl: Methylotrophic reactor). An OTU is considered cultivated if it grew in either of the two bioreplicates. c) Cultivation success at the OTU level across autotrophic and heterotrophic conditions for droplet- and plate-based cultivation methods. Success was defined by the growth status of the most abundant ASV within each OTU. Boxplots show the percentage of OTUs meeting this criterion for each method–condition combination, with points representing individual enrichment culture datapoints. Colors indicate method–condition combinations, and point shapes represent different enrichment types.

We also compared the overall cultivation success of droplet- and plate-based methods by calculating, for each bioreplicate and enrichment culture, the percentage of OTUs in the reference inoculum whose most abundant ASV grew (Fig. 2c). In autotrophic conditions, plates achieved a slightly lower median cultivation rate than droplets (68.4 ± 2.1% vs. 70.7 ± 7.9% per enrichment; mean difference (d-p) = 4.1%, paired one-sided bootstrap test, *P* = .13, Bonferroni-adjusted *P* = .25). A similar but more significant pattern was observed in heterotrophic conditions, where plates cultivated only 51.7 ± 2.6% of OTUs on median, compared to 77.8 ± 8.2% for droplets (mean difference (d-p) = 28.9%, paired one-sided bootstrap test, *P* < .001, adjusted *P* = .001).

### Scaled to inoculum size, droplets outperform plates in median cultivation success

To isolate the effect of the cultivation method, we controlled for differences in sampling depth between methods. We included only OTUs with a high chance (≥95%) of being detected by the method with the lowest number of inoculated cells. This chance was calculated from each OTU’s relative abundance in the inoculum and the number of cells inoculated, giving a common set of OTUs for comparison (Table 1). A detailed description of this probability-based filtering approach is provided in SI section 1.1.2.2. Using this subset, we compared droplet- and plate-based cultivation under autotrophic and heterotrophic growth conditions by calculating, for each enrichment type, the proportion of OTUs for which the most abundant ASV was successfully cultured.

**Table 1:** The number of inoculated cells at the start of each experiment for each method, condition, and enrichment source community.

Condition	Enrichment source community	Droplets	Plates	Relative frequency threshold
Heterotrophic conditions	Hydrogen reactor	80 000	1000	0.003
	Aerobic sludge	80 000	1000	0.003
	Methylotrophic reactor	80 000	10 000	0.0003
				
Autotrophic conditions	Hydrogen reactor	80 000	10 000	0.0003
	Aerobic sludge	80 000	10 000	0.0003
	Methylotrophic reactor	80 000	10 000	0.0003

The relative frequency threshold used to define detectable OTUs (≥95% probability of inclusion in the method with the smallest sampling effort) is reported in the final column.

Across all enrichment types (*n* = 6 for heterotrophic conditions, *n* = 4 for autotrophic conditions), both methods yielded high cultivation success under autotrophic conditions, with median values above 85% (Fig. 3). Droplet-based autotrophic cultivation showed a slightly higher median than plate-based autotrophic cultivation and exhibited higher variability between samples (droplet: 92.3 ± 10.4%, plate: 89.5 ± 6.3%, mean difference (droplets-plates) = −0.26%, paired one-sided bootstrap p = 0.62, Bonferroni-adjusted *P* = 1.00). Several droplet assays and one plate replicate achieved complete recovery of the considered OTUs.

**Figure 3:**
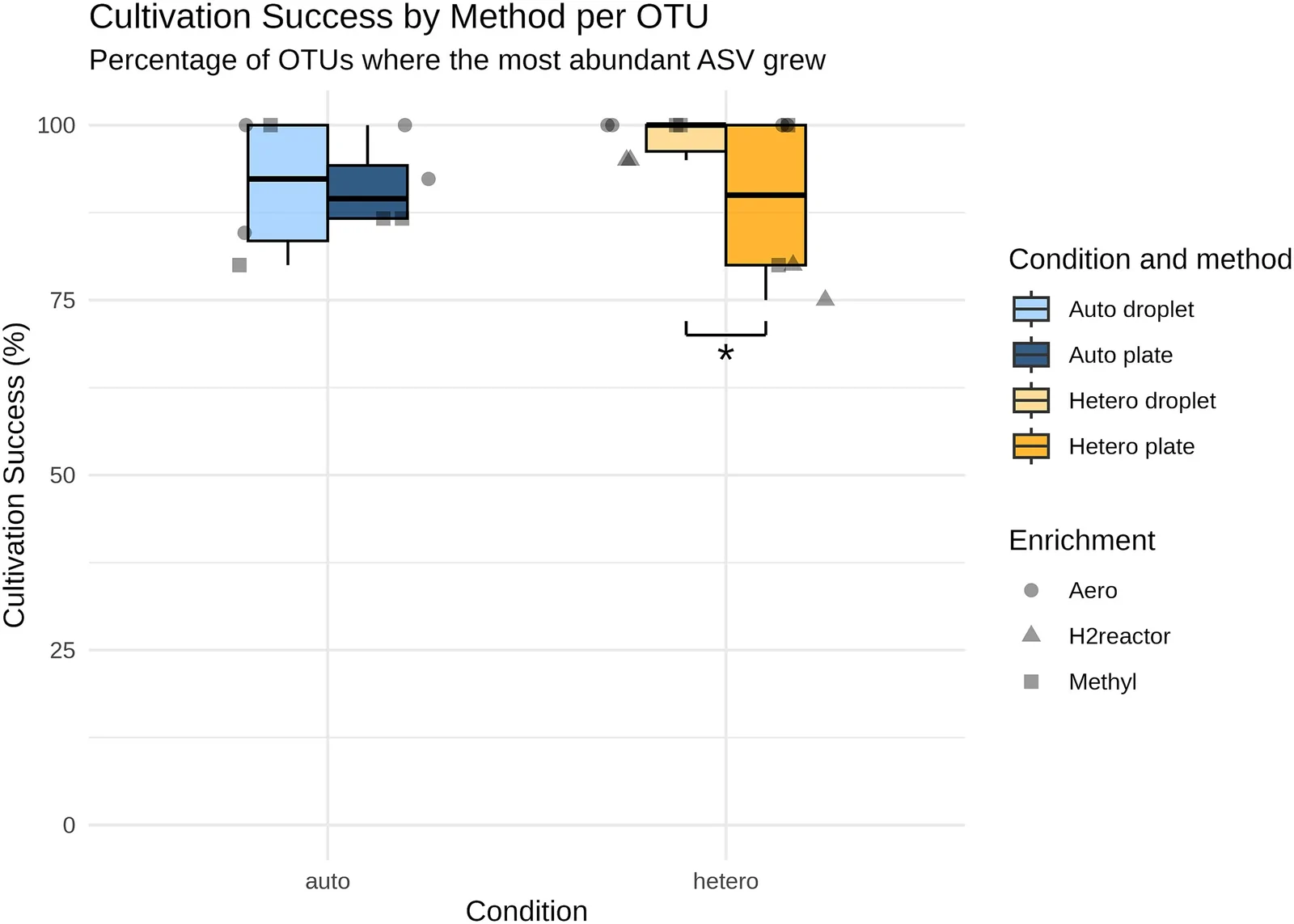
Cultivation success at the OTU level across autotrophic and heterotrophic conditions for droplet- and plate-based cultivation methods, restricted to OTUs meeting the 95% inclusion criterion. Success was defined by the growth status of the most abundant ASV within each OTU. Boxplots show the percentage of these OTUs meeting this criterion for each method–condition combination, with points representing individual enrichment culture datapoints. Colors indicate method–condition combinations, and point shapes represent different enrichment types.

Under heterotrophic conditions, both droplet- and plate-based methods achieved high recovery rates, with medians comparable to those observed in the autotrophic treatments. The heterotrophic droplet method had a significantly higher median than plates (mean difference of medians (droplets-plates) = 9.2%, *P* = .019, Bonferroni-adjusted *P* = .037) and relatively tight distributions across enrichment types (100.0 ± 2.6%), indicating stable performance (Fig. 3). The plate-based method showed high recovery with greater variability (90.0 ± 12.0%), ranging from full OTU recovery in some cases to values below 80% in others. No significant difference was detected for autotrophic conditions, where plates cultivated, on average, 89.5 ± 6.3% of eligible OTUs and droplets cultivated 92.3 ± 10.4% (mean difference of medians (droplets-plates) = −0.26%, *P* = .6235, Bonferroni-adjusted *P* = 1). For completeness, an OTU-level full list of taxa that were not cultivated by either cultivation method or by one of the two methods, in at least one replicate, is provided in Table S2 (SI).

### Rare OTUs tend to be more effectively cultivated by droplets

To assess whether differences in cultivation success are influenced by the rarity of a strain, we grouped OTUs by their relative abundance in the inoculum and examined cultivation trends per method (Fig. 4). For autotrophic conditions (*n* = 4), the bin representing 0.1–1% abundance showed significant differences in cultivation success with droplets outperforming plates (mean difference = 15, *P* = .002, BH-adjusted *P* = .01; paired bootstrap resampling), but with the reverse, non-significant trend seen for the lowest bin 0.01%–0.1% (mean difference = −15.8, *P* = .80, adjusted *P* = .999; paired bootstrap). Under heterotrophic conditions (*n* = 6), droplet recovery averages outperformed plates in two of the lowest abundance bins, 0.01%–0.1% (mean difference = 25, all differences positive but with no variability, so no significance called) and 0.1%–1% (mean difference = 15, *P* = .056, adjusted *P* = .14; paired bootstrap). Taken together, these results show a trend in which droplets tend to outperform plates in OTU recovery for the lowest abundance bins.

**Figure 4:**
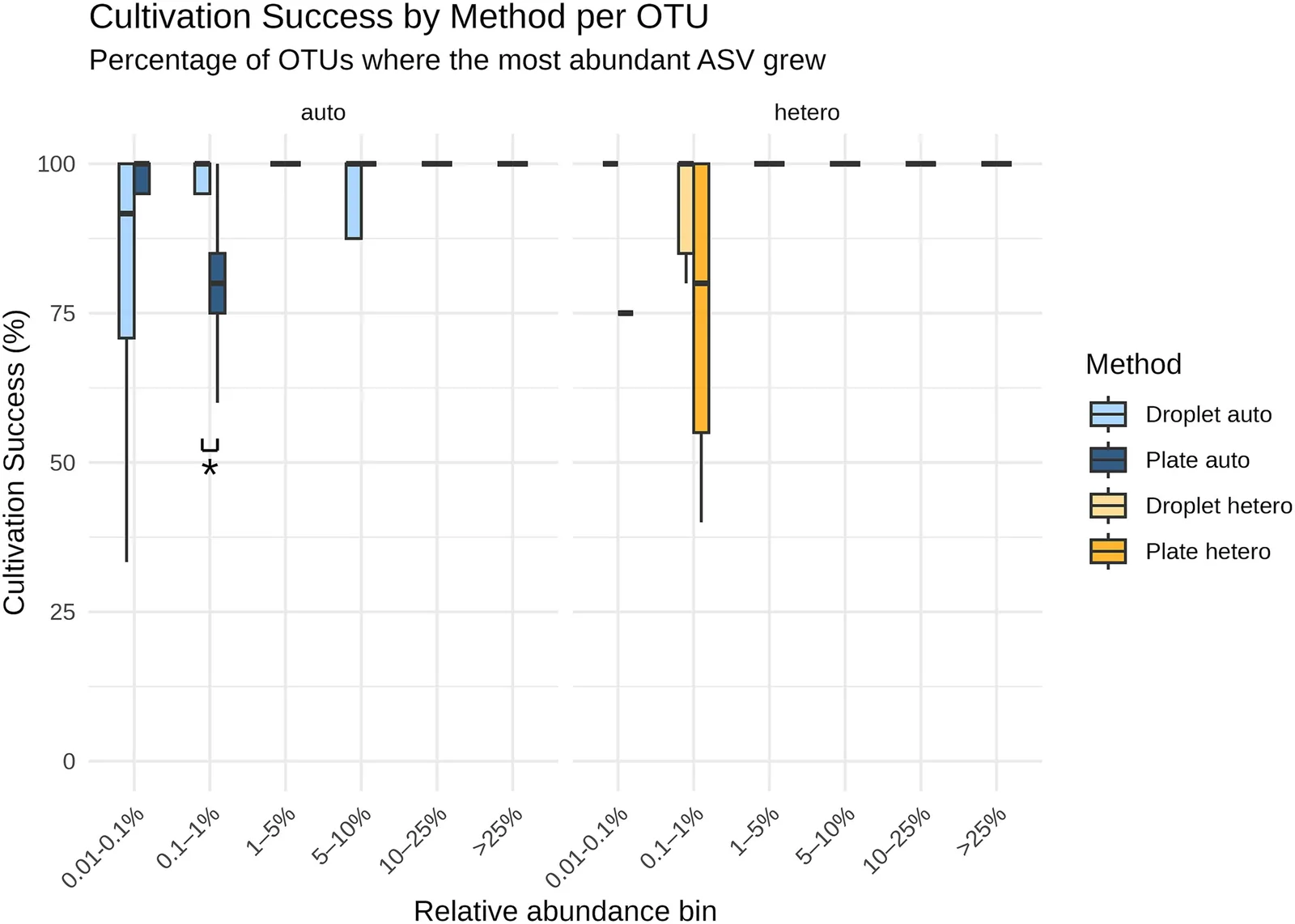
Cultivation success at the OTU level per relative abundance bin in the inoculum. Determined across autotrophic and heterotrophic conditions for droplet- and plate-based cultivation methods, restricted to OTUs meeting the 95% inclusion criterion. Success was defined by the growth status of the most abundant ASV within each OTU. Colors indicate method–condition combinations. No significance is shown for the 0.01–0.1% bin for the heterotrophic condition because all paired differences were positive but identical, preventing estimation of variance.

### Droplet growth reflects resource compartmentalization

Because competitive overgrowth can lead to unequal expansion among taxa, we examined whether OTU growth was more even in droplets than on plates, as would be expected if compartmentalization constrained competitive dominance. To minimize taxonomic bias, we restricted the analysis to OTUs that grew in both cultivation methods. For these shared OTUs, fold changes were median-normalized, and the IQR was calculated as a measure of variability in OTU expansion. A lower IQR indicates that OTUs showed more similar fold changes, and therefore greater growth evenness, whereas a higher IQR indicates more unequal expansion among OTUs.

Across both autotrophic and heterotrophic conditions, the paired differences in IQR showed the same trend, with droplets showing more even growth compared to plates, although neither comparison reached statistical significance (Fig. 5, SI: Fig. S10). For autotrophic conditions we saw that the mean paired difference of IQR between the two methods (droplets-plates) was −3.76 (*n* = 4, *P* = .18, paired Wilcoxon, Bonferroni-adjusted *P* = .36). For heterotrophic conditions we saw that the mean paired difference of IQR between the two methods (droplets-plates) was −16.64 (*n* = 6, *P* = .26, paired Wilcoxon, Bonferroni-adjusted *P* = .52).

**Figure 5:**
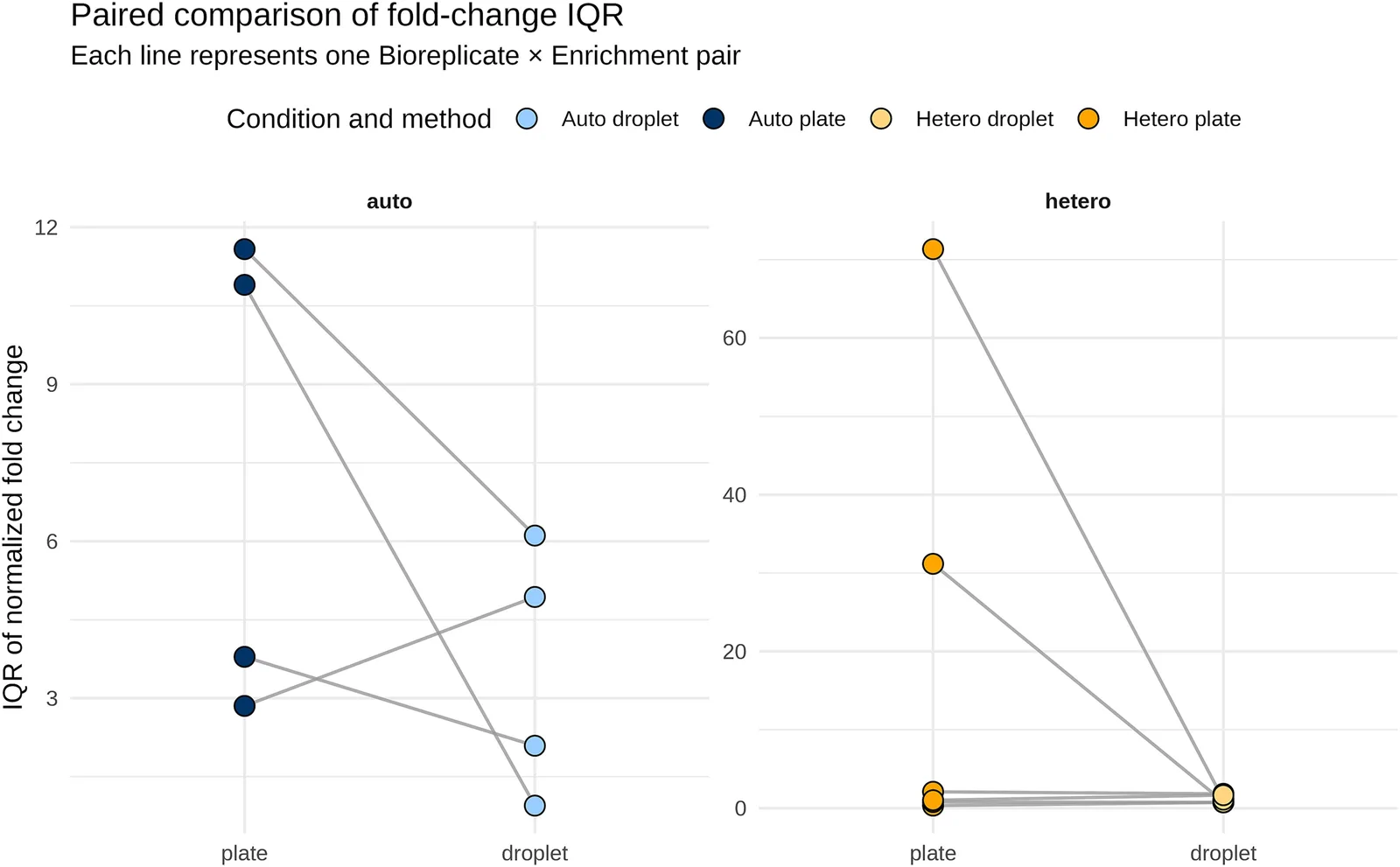
Droplet cultivation tended to produce more similar OTU fold changes than plate cultivation, where some OTUs expand more strongly than others. Variability in median-normalized OTU fold changes between plate-based and droplet-based cultivation is expressed using interquartile range (IQR) of normalized OTU fold changes for each enrichment x biological replicate under autotrophic and heterotrophic conditions. Plate and droplet values from the same enrichment and replicate are connected by gray lines. Lower IQR values indicate a more even distribution of fold changes among OTUs, whereas higher values indicate greater variability in the extent of OTU growth.

To further support this interpretation, we investigated whether the growth-limiting nutrient (NH_4_^+^) remained confined within individual droplets. Although cells are physically separated, molecular cross-talk could allow nutrients to move between droplets, potentially weakening resource compartmentalization. We therefore compared biomass yield per occupied droplet under low- and high-occupancy autotrophic conditions (Low: 3.9% droplets with at least one cell, high: 86.5% with at least one cell). If NH_4_^+^ freely crossed between droplets, low-occupancy cultures should support more cells per occupied droplet because NH_4_^+^from the many empty droplets would become available. In this case, there is roughly 22.2-fold more NH_4_^+^ available in the low-occupancy condition compared to the high-occupancy condition. After cultivation, droplets were coalesced, SYBR Green-stained, and counted by flow cytometry, while droplet numbers were estimated from the recovered droplet volume. Cell numbers per occupied droplet did not consistently increase at low occupancy (Aero: 50.0 ± 14.1 (low) vs. 44.4 ± 15.7 (high); Methyl: 36.7 ± 4.7 (low) vs. 55.5 ± 7.9 (high) cells per droplet; Table 2, SI: Table S3), providing no evidence for cross-droplet transfer of NH_4_^+^ enabling cross-droplet competition. Rather than showing the ~22-fold increase expected under complete nutrient cross-talk, low- and high-occupancy droplets produced similar cell yields. This indicates that growth was largely supported by resources retained within individual droplets.

**Table 2:** Expected vs. observed cell yield under complete nutrient cross-talk.

Enrichment	Observed high-occupancy cells/droplet	Expected low-occupancy cells/droplet if fully leaky	Observed low-occupancy cells/droplet
Aero	44.4 ± 15.7	~985	50.0 ± 14.1
Methyl	55.5 ± 7.9	~1231	36.7 ± 4.7

Expected low-occupancy cell numbers were calculated by scaling the observed high-occupancy cell yield by the 22.2-fold difference in theoretical nutrient availability between 3.9% and 86.5% occupancy, assuming biomass yield scales directly with access to the limiting nutrient and that nutrients are freely redistributed between droplets. Observed low-occupancy cell yields were substantially lower than these theoretical values, providing no evidence for cross talk of limiting nutrient between droplets.

Although yeast extract in the heterotrophic medium could introduce additional cross-talk nitrogen compounds, both majority of nitrogen in both media consisted of the same basal ammonium supply (4 g/l NH_4_Cl); therefore, retention of the limiting nutrient under autotrophic conditions supports comparable nutrient confinement in the heterotrophic experiments, despite the additional nitrogen contributed by yeast extract.

## Discussion

Isolation methods continue to further our understanding of microbial diversity and function. Here, we compared droplet cultivation with plate cultivation to recover bacteria of three different enrichments in two different media to better understand the method-specific effects of droplet cultivation.

Throughput normalization had a large effect on the mean performance difference between the two methods in heterotrophic conditions, falling from 28.9% (throughput-uncorrected, droplets-plates) to 9.2% (throughput-corrected, Figs. 2 and 3). The effect of cultivation throughput on taxon recovery is conceptually expected: increasing the number of inoculated cells increases the number of taxa that are sampled from the inoculum. However, we argue that making this distinction is especially relevant for droplet cultivation, for which high throughput is a major intrinsic methodological advantage and the contribution of which is not always separated from cultivation-specific effects in literature reports (Table 3). As a result, greater taxonomic recovery in these reports can be interpreted as evidence that taxa grow more successfully in droplets, even when part of the observed advantage may simply arise because deeper screening increases the number of taxa that are included in the experiment.

**Table 3:** Comparison of reported cell and sequencing numbers from droplet- and plate-based studies.

Paper	Starting environment	Plate inoculated number of cells	Droplets inoculated number of cells	Ratio (D/P)
Yin *et al.,* 2022	Fecal community	6000	6000	1x
Mahler *et al.*, 2021	Soil community	300–3000	3.6 × 10^6^	~1000x–10.000x
Watterson *et al.*, 2020	Fecal community	15 000 000	750 000	0.05x
Seo *et al.,* 2026	Soil community	5000	9.6 × 10^6^	~ 2000x

Ratio (D/P) represents the ratio of droplet founder cells over plate founder cells. For Mahler (2021), droplet founder cell counts were estimated from reported values: 9 × 10^6^ droplets of 200 pl (=1.8 mL total), and the reported droplet occupancy corresponds to ~2 × 10^6^ cells/ml (soil community density), totaling 3.6 × 10^6^ founder cells sequenced (one droplet population). 50 μl of 1:100 and 1:1000 dilution of the soil community was plated. For Seo *et al.* (2026), droplet founder-cell numbers were estimated from the reported 600 μl sample volume, an average agarose bead diameter of 20 μm, and an assumed random packing fraction of 0.67. These values correspond to ~9.6 × 10^7^ agarose beads; with ~10% of beads reported to contain a single cell, this yields ~9.6 × 10^6^ founder cells in the droplet population. All other values are taken directly from the cited papers. More details about how these calculations were done can be found in SI.

Previous studies, all reporting increases in number of cultivated taxa compared to plates, illustrate how these effects have become entangled. Mahler *et al.* (2021) and Seo *et al.* (2026) screened substantially more founder cells in droplets than on plates [18, 23], whereas Yin *et al.* (2022) used comparable founder-cell numbers and Watterson *et al.* (2020) screened more cells on plates [20, 21]. Droplet-associated increases in recovery (compared to plates) have been reported across these different designs. This indicates that an increased throughput alone cannot explain the droplet advantage observed across the literature (i.e. reports on cultivation in droplets have shown droplet-specific effects that increase the number of recovered taxa compared to plates [20, 21]), but it can substantially inflate its apparent magnitude in individual comparisons with other more low-throughput techniques. We therefore identify unequal sampling depth as an underappreciated source of bias in comparisons of droplet- and plate-based cultivation. This distinction is important because comparisons based on raw taxonomic recovery may overstate the extent to which the droplet environment itself improves growth.

We hypothesized that stronger physical separation in droplets reduces interspecies competition. Two observations indicate that our droplet system had the properties required to constrain competitive overgrowth. First, growth-limiting nutrients appeared to remain largely confined within individual droplets: despite a >20-fold theoretical difference in nutrient availability per occupied droplet under assumed complete cross-talk, per-droplet cell yields did not systematically increase under low-occupancy conditions (Table 2). Second, OTU fold changes tended to be more evenly distributed (autotrophic mean paired difference of IQR (plate-droplets) = −3.76, heterotrophic = −16.64). in droplets than on plates across both cultivation conditions (Fig. 5), indicating less extreme differences in population expansion. Together, these observations suggest that droplets functioned as effective resource compartments and limited the extent to which strongly growing populations could compete.

These observations are consistent with previous work showing that droplet compartmentalization can reduce competitive dominance. Bachmann *et al.* showed that resource privatization in emulsions shifted selection away from rapid growth toward slower-growing, higher-yield strategies [32], McCully *et al.* showed that droplet compartmentalization can limit domination of fast-growing taxa and favor slower-growing populations [33]. The effect of compartmentalization removing competition is unlikely to be absolute across all droplet cultivation efforts, however, because droplets can exhibit molecular cross-talk between compartments depending on the medium compounds and oil and surfactant used [34–36].

The second part of our hypothesis was that reduced competitive dominance would support detectable growth of a broader range of taxa. After correcting for throughput, a residual droplet advantage of 9.2% remained under heterotrophic conditions, whereas little or no clear advantage remained under autotrophic conditions (Figs. 2 and 3). Thus, screening depth did not fully explain the difference between methods under heterotrophic conditions. Within this residual effect, the droplet advantage tended to be concentrated among lower-abundance OTUs (Fig. 4). In our enrichment system, repeated growth and dilution are expected to enrich taxa with greater realized growth under the applied conditions, while more slowly expanding or competitively disadvantaged taxa may remain at lower abundance. If part of the rare fraction therefore consisted of slower-growing taxa, their preferential recovery in droplets would be consistent with competitive release since they would benefit disproportionately from reduced competitive dominance^51,52^ in droplets, potentially explaining the rarity-dependent recovery trend.

When ignoring the rarity-dependent trend, the residual difference in taxonomic recovery does not require a competition-based explanation. Droplets and plates also impose fundamentally different growth modes: cells grow planktonically in liquid droplets but as surface-associated colonies on solid medium. Surface attachment is known to trigger substantial physiological and regulatory changes relative to planktonic growth [37], and liquid- and solid-based cultivation have been shown to recover different fractions of microbial communities [38–40] . Because our enrichment communities were maintained in liquid culture, they may also have been biased toward taxa performing well under planktonic growth, potentially favoring recovery in droplets. Other differences, including exposure to surfactant in droplets, gas transfer, diffusion distances, local metabolite accumulation, the plate gelling agent, and exposure to shear during droplet generation, may also contribute to the residual method effect. Thus, part of the remaining droplet advantage may reflect physiological responses to the cultivation format itself rather than competitive release alone.

### Limitations of the study

This study used three independently derived enrichment communities; each tested in biological duplicate. We prioritized multiple enrichments to broaden the taxonomic scope of the comparison, while anticipating relatively large differences between droplet- and plate-based cultivation. Replication was constrained by the technical complexity and sample-handling demands of the droplet workflow. Because the observed method effects were more modest than expected, some secondary analyses, particularly abundance-dependent recovery and growth-evenness comparisons, were consequently underpowered.

A second limitation is that the study was performed on enrichment communities, enriched with fast-growing bacteria (subculturing happened twice weekly) rather than unprocessed environmental samples where slower-growing K-strategists are more likely to be present. This provided a controlled system for comparing cultivation formats but also reduced ecological complexity and selected for organisms already adapted to the applied media and repeated transfer regime. Consequently, the high percentage of OTUs isolated, the magnitude of the observed droplet advantage, and the rarity dependence effect of recovery cannot be assumed to generalize directly to natural microbial communities.

## Conclusion

Our study demonstrates that high recovery of microbial richness from hydrogen-oxidizing enrichments is achievable using both droplet- and plate-based cultivation methods, with combined recovery rates approaching 90% of observed OTUs under both autotrophic and heterotrophic conditions. Droplet cultivation recovered more OTUs overall, but much of this apparent advantage was explained by its higher throughput. After accounting for screening depth, a residual droplet advantage remained under heterotrophic conditions, whereas no clear advantage remained under autotrophic conditions. Our working hypothesis was that stronger physical separation in droplets reduces interspecies competition and thereby supports detectable growth of a broader range of taxa. The retention of growth-limiting resources within droplets and the tendency toward more even OTU expansion were consistent with effective compartmentalization and reduced competitive dominance, while the greater recovery of lower-abundance OTUs provided suggestive support for competitive release. Comparison with previous droplet-cultivation studies further showed that differences in throughput are not always separated from cultivation-specific effects, potentially confounding the mechanistic interpretation of increased recovery frequently reported in droplet cultivation literature. Together, our results indicate that the apparent advantage of droplet cultivation reflects both greater screening depth and cultivation-format-specific effects and highlight the importance of distinguishing these contributions when evaluating and applying high-throughput cultivation methods.

## Supplementary Material

Supplementary_materials_ycag289

## Data Availability

All data underlying the results presented in this study, as well as the scripts used to generate the figures, are publicly available at https://osf.io/52c9z/overview?view_only=f234ee877033404980d4ec96a9f6bb25.
