# Supplementary material for: Throughput and compartmentalization shape bacterial recovery in droplet- and plate-based cultivation of hydrogen-oxidizing communities
Source: ISME Commun. 2026 Oct 9;6(1):ycag289. doi: 10.1093/ismeco/ycag289 (PMC13747019; doi:10.1093/ismeco/ycag289)
Supplement: Supplementary file 1 [file supplementary_materials_ycag289.docx]

Throughput and compartimentalization shape bacterial recovery in droplet- and plate-based cultivation of hydrogen-oxidising communities - Supplementary information

## 1.1 Supplementary Materials and methods

### 1.1.1 Liquid and solid media preparation and detailed enrichment procedure

Mineral DSMZ-81 medium was prepared according to DSMZ specifications with several modifications. Three solutions were made: solution A was made by dissolving 2.3 g KH_2_PO_4_ and 2.9 g Na_2_HPO_4_·2H_2_O in 50 mL distilled water. Solution B was made by dissolving the following in 920 mL distilled water: 4.0 g NH_4_Cl (increased from 1.0 g), 0.5 g MgSO_4_·7H2O, 10 mg CaCl_2_·2H_2_O, 5 mg MnCl2·4H_2_O, 5 mg NaVO_3_·H_2_O, and 5 mL trace element solution SL-6. SL-6 is made of 0.1 g/L ZnSO_4_·7H_2_O, 30 mg/L MnCl_2_·4H_2_O, 0.3 g/L H_3_BO_3_, 0.2 g/L CoCl_2_·6H_2_O, 10 mg/L CuCl_2_·2H_2_O, 20 mg/L NiCl_2_·6H_2_O, and 30 mg/L Na_2_MoO_4_·2H_2_O in distilled water. Solution C was altered: 50 mg FeSO_4_·7H_2_O and 60 mg Na_2_EDTA·2H_2_O were dissolved in 20 mL distilled water. We altered this solution to avoid introducing bioavailable organic carbon from citrate which was used in the original recipe. Solutions A, B, and C were autoclaved separately for 15 min at 121 °C. All solutions were combined aseptically together with 10 mL/L 50 g/L NaHCO_3_ solution. No vitamin solution was added. The final solution was filter sterilised and stored at room temperature. All aforementioned chemicals were bought from Sigma-Aldrich (Merck KGaA, Darmstadt, Germany).

For heterotrophic conditions, yeast extract (Carl Roth, Karlsruhe, Germany), pyruvic acid (Sigma-Aldrich, Merck KGaA, Darmstadt, Germany), and glucose (Sigma-Aldrich, Merck KGaA, Darmstadt, Germany), were prepared as 200 g/L stock solutions in distilled water and filter-sterilised. Filter sterilised mineral DSMZ-81 medium (prepared as described above) was supplemented with these stock solutions to final concentrations of 0.2% (w/v) yeast extract, 0.6% (w/v) pyruvic acid, and 0.6% (w/v) glucose. The final solution was stored at room temperature. This formulation is referred to as rich DSMZ-81, whereas the unsupplemented medium is referred to as mineral DSMZ-81.

To prepare solid medium, Gelzan™ (gellan gum, Sigma-Aldrich, Merck KGaA, Darmstadt, Germany) was used as a more recalcitrantalternative to agar. Gellan gum (10 g/L) was added to solution B and autoclaved; the remaining solutions were added aseptically while hot to prevent gelling. Plates were poured under aseptic conditions.

Three hydrogen-oxidising enrichment communities were established from a methylotrophic reactor, a hydrogenated trickling filter reactor[1] and post-aeration sludge from a water treatment plant (RWZI, Gent, Belgium). These environments were chosen due to their promising potential for harbouring aerobic hydrogen-consuming bacteria, serving as a good starting point for the enrichments. The samples (3 mL) were vortexed to dislodge biofilm into planktonic cells after which they were centrifuged (5000xg for 5 minutes). They were washed once with 3 mL 1x PBS (Sigma-Aldrich, Merck KGaA, Darmstadt, Germany), after which they were diluted to an OD600 of 0.05 in 5 mL mineral DSMZ-81 medium. These inocula were added to sterile Hungate tubes that contained a gas atmosphere consisting of 73% H_2_, 18% CO_2_ and 9% O_2_. The tubes were incubated at 28 °C with shaking at 120 rpm. Growth was monitored by observing turbidity and measuring pressure. Once a significant pressure drop was detected – from an initial pressure of approximately 125 kPa to below 90 kPa – the cultures were diluted 1:10 in mineral DSMZ-81 medium (5 mL total volume). Over the four-week enrichment period, the cultures developed stable growth, with dilutions carried out twice weekly. Upon completion of the enrichment process, the headspace was aseptically refreshed to maintain stable conditions during storage. The enriched cultures were stored at 4 °C until needed for subsequent experiments.

#### 1.1.1.1 Inoculum preparation

Enrichment cultures stored at 4 °C were diluted 1:10 in mineral DSMZ-81 medium and incubated overnight under autotrophic conditions (73% H_2_, 18% CO_2_, 9% O_2_) at 28 °C with shaking at 120 rpm. Cultures were harvested during exponential growth, as indicated by cell counts and a measurable pressure drop in the headspace. Cells were pelleted by centrifugation (5000xg, 3 min), and the supernatant was discarded. Pellets were washed three times with ice-cold DSMZ-81 medium to remove residual carbon sources, with all handling performed on ice to minimise metabolic activity and cellular stress. The washed cell pellet was resuspended in fresh DSMZ-81 medium to create a concentrated cell stock, which was then diluted in the appropriate medium to achieve the desired starting concentration for experiments.

#### 1.1.1.2 Droplet cultivation

The starting inoculum was diluted to 2×10^6^ cells/mL with heterotrophic conditions being diluted with rich DSMZ-81 and autotrophic conditions being diluted with mineral DSMZ-81; for 20 pL droplets this corresponds to a Poisson parameter of λ = 0.04, yielding ~4% single-cell occupancy (P_Poisson_(X=1) = (0.04^1^*e^-0.04^)/(1!) = 3.8%), with the remainder of the droplet predominantly empty. Droplets were generated by flowing the inoculum and immiscible surfactant/oil mixture (HFE7500 (3M, Zwijndrecht, Belgium) + 0.5 w/w% Picosurf (Sphere Fluidics, Royston, UK) into a 20 µm height T-junction droplet generation chip with inoculum syringes kept on ice to prevent cellular growth (to prevent skewing the Poisson distribution).. Droplet volume (20 pL) was calculated using the aqueous flow rate and droplet production frequency. For detailed operational parameters, see SI: Single emulsion production. After collection, 600 µL of droplets were mixed with 1500 µL HFE7500 + 0.1 w/w% Picosurf and transferred into a Novec 1720-treated (3M, Zwijndrecht, Belgium) Hungate tube. For heterotrophic samples, 2 mL of the droplet:oil mixture was transferred into sterile 2 mL Eppendorf tubes, maintaining the same droplet-to-oil-ratio as used for the autotrophic droplets. Following loading into their respective containers, droplets were incubated for 10 days while shaking at 120 rpm at 28 °C and were subsequently stored at 4 °C until sample processing. All experiments were performed in biological duplicate. Bacterial growth within droplets was assessed by microscopy (cell morphology and active motility) and independently validated by SYBR Green staining combined with flow cytometry (see SI, Figure S 20). For sample processing, droplets were coalesced using perfluoro-1-octanol (PFO; Sigma–Aldrich, Merck KGaA, Darmstadt, Germany) in a fume hood. Two microliters of the coalesced droplets were aspirated for flow cytometric counts, and two aliquots of 40 µL were frozen at -20 °C in DNA extraction tubes containing beads for later DNA extraction.

#### 1.1.1.3 Plate cultivation

Inoculum was diluted tenfold up to 10^-6^, and 100 uL of the dilutions 10^-3^ to 10^-6^ were plated on gellan gum mineral DSMZ-81 plates or gellan gum rich DSMZ-81 plates for autotrophic and heterotrophic conditions, respectively. To enable autotrophic growth, mineral DSMZ-81 plates were incubated in a gas jar (3.5 L Oxoid, Thermo Fisher Scientific, Basingstoke, UK) with a custom atmosphere (73% H_2_, 18% CO_2_, 9% O_2_). To obtain this custom atmosphere, the gas jar was flushed for 15 minutes at a flow rate of 4 L/min with an 80%H_2_:20%CO_2_ gas mixture (Air Liquide, Paris, France). After flushing, 350 mL pure O_2_ was added with a 50 mL syringe. All gas flushing and gas addition was done through a 0.2 µm filter. Plates were incubated at 28 °C for 8 days. All procedures were performed in biological duplicate. Samples for DNA extraction and flow cytometric counts were gathered by adding 3 mL filter sterilised PBS to a plate and scrubbing the colonies from the plate surface with a cell spreader. Samples for DNA extraction were spun down at 13.000 g for 1 minute. Samples for determining cell counts were fixed by adding 1 uL glutaraldehyde (20% v/v) to 250 µL sample[2] and stored at 4 °C for later flow cytometric analysis.

#### 1.1.1.4 Microfluidic device fabrication

Microfluidic designs were created in AutoCAD 2022 (Autodesk Inc., USA). Silicon wafers were fabricated using standard photolithography with 10 µm or 20 µm relief heights. PDMS (Sylgard 184, Dow) was mixed with curing agent (10:1 mass ratio), poured onto SU-8 moulds, degassed under vacuum cycles, and cured overnight at 60 °C. The cured PDMS was demoulded, channels cut, and inlet/outlet holes punched (1.25 mm biopsy punch, WPI, USA), then cleaned with isopropanol (Carl Roth) and dried with pressurised air. Surfaces of PDMS chips and glass slides (Carl Roth, Belgium) were cleaned with Scotch tape (3M, Belgium) and treated with oxygen plasma (12 s, 100 W, 20 sccm O_2_, 0.65 mbar; Femto Plasma System, Diener Electronic). PDMS and glass were aligned and bonded; bonding was verified by attempting separation.

For hydrophobic chips, Novec 1720 (3M, Belgium) was introduced into channels via micropipette, evaporated in a desiccator (~15 min), and baked at 135 °C for 15 min. Inlets were sealed with Scotch tape for storage at room temperature.

For water-in-oil emulsion imaging, a PDMS droplet observation chamber was cast over a cover slip, degassed, baked, cut, punched, plasma-treated, and hydrophobised as above, then bonded to a large cover slip (50 × 22 mm, Fisher Scientific).

#### 1.1.1.5 Single emulsion production

Water-in-oil emulsions were generated using two syringe pumps (TSE systems type 540060). Syringes were connected to the PTFE tubing (1/16” OD, 0.3 mm ID, Deutsch & Neumann, Germany) using Luer lock-HPLC fittings (IDEX Health and Science, USA). The standard flow rates used were 450 µL/hr aqueous phase and 500 µL/hr oil phase, to decrease droplet size oil flow rate was increased. Microfluidic operations were monitored using an inverted microscope (Zeiss Axiovert 135) and footage was recorded using a high-speed camera (S-Motion Mono 2.6 GB High Speed Camera, AOS Technologies AG). All droplet populations were around 20 pL in volume, for these, droplet chips were used that are 20 µm in height. For all droplet samples, the oil phase consisted of 0.5 w% Pico-Surf (Sphere Fluidics) in HFE-7500.

#### 1.1.1.6 Microscopy

Single emulsion populations were imaged in a droplet chamber filled with HFE-7500, into which ~2 µL emulsions were added. PDMS plugs sealed both outlets, and the chamber was incubated on its side for 2 min to form a droplet monolayer, then placed coverslip-side up on the imaging platform. All imaging was performed using an Axioskop 2 plus microscope (Zeiss) with a QImaging Retiga R3 CCD camera (Cairn Research).

#### 1.1.1.7 Flow cytometry

Samples from scraped plate colonies (resuspended in PBS) and coalesced droplets were diluted in 0.2 µm–filtered PBS, stained with 1× SYBR Green I (Invitrogen, USA) for 20 min at 37 °C in the dark, and analysed on an Accuri C6+ flow cytometer (BD Biosciences, USA) to determine total cell concentration (fast settings). Double emulsions were made and stained as described here[3] after which they were analysed on an Accuri C6+ with slow flow rate settings.

#### 1.1.1.8 DNA extraction, library preparation and sequencing

DNA extraction of the collected cell biomass was performed using the DNeasy PowerSoilPro kit (Qiagen, Germany). This genomic DNA extract was sent out to VIB Nucleomics Core (Leuven, Belgium) for Kinnex library preparation and sequencing on a PacBio Revio platform. In short, genomic DNA was quantified using a Qubit fluorometer (Thermo Fisher Scientific). DNA was normalised and amplified with PCR using barcoded Kinnex primer pairs (Forward: 27F: 5′-GCATC/[forward_barcode]/AGRGTTYGATYMTGGCTCAG-3′, Reverse: 1492R: 5′-GCATC/[reverse_barcode]/RGYTACCTTGTTACGACTT-3′). The barcoded amplicons were pooled, annealed, and concatenated, then converted into SMRTbell libraries according to the “Preparing Kinnex Libraries from 16S rRNA Amplicons v2024.07” protocol. Libraries were sequenced on a PacBio Revio platform.

Raw HiFi reads were processed using PacBio SMRT Link to generate CCS reads. Concatenated amplicons were de-concatenated with Skera v2024.07. Demultiplexing, primer removal, and length filtering (retaining reads >50 bp) were performed with Lima v2.5.0, assigning reads to samples based on dual barcode sequences[4].

#### 1.1.1.9 Bioinformatics pipeline

Raw reads were processed using the Pacific Biosciences HiFi-16S-workflow[5]. In short; reads were quality-filtered (mean QV ≥ 20) and length-gated to 1000-1600 bp. Primers were trimmed and read orientation corrected with cutadapt using 27F (AGRGTTYGATYMTGGCTCAG) and 1492R (AAGTCGTAACAAGGTARCY)[6]. Amplicon sequence variants were inferred with DADA2 using the PacBio error estimation function (PacBioErrfun; maxEE = 2, minQ = 0; pseudo-pooling), including dereplication and chimera removal[7]. Parameters followed the PacBio HiFi-16S workflow defaults. Obtained ASVs were classified using the silva138.1 database[8].

#### 1.1.1.10 Estimation of founder-cell numbers in previous studies.

Where founder-cell numbers were not reported directly, they were reconstructed from the experimental parameters provided by the authors. For Mahler *et al.* (2021) [9] , droplets were generated from the undiluted suspension referred to as the “soil community”. The reported Poisson loading parameter (lambda = 0.4) and droplet volume (200 pL) correspond to an estimated cell concentration of 2*10^6^ cells/mL consistent with the reported droplet occupancy of approximately 30%. With 9*10^6^ droplets generated, corresponding to 1.8 mL of aqueous droplet volume, this yields approximately 3.6*10^6^ founder cells in one droplet population. Because plate cultivation used 50 µL of 1:100 and 1:1000 dilutions of the same soil-community suspension, the same estimated starting concentration was used to calculate approximately 1,000 and 100 founder cells plated, respectively.

For Seo *et al.* (2026), founder-cell numbers were estimated from the reported 600 µL sample volume and agarose microdroplet (GMD) characteristics. GMDs ranged from 10-30 µm in diameter; an average diameter of 20 µm was therefore used together with an assumed random packing fraction of 0.67 [10] , yielding an estimated 9.6*10^7^ GMDs in the processed sample [11] . The authors reported that approximately 8-12% of GMDs contained a single microbial cell; using the midpoint of 10% gives an estimated 9.6*10^6^ founder cells in the droplet population (using Poisson distribution statistics with lambda = 0.098, and droplet size 4.1 pL). For the corresponding plate cultivation, the reported inoculum contained approximately 5,000 founder cells. These calculations should be regarded as approximate estimates of sampling depth rather than exact founder-cell counts.

### 1.1.2 Data processing and analysis

#### 1.1.2.1 General

Rarefaction was applied to account for differences in sequencing depth. For Part 1 of the study, where inocula were compared directly, samples were rarefied across all inocula to enable consistent richness comparisons. In Parts 3 and 4, rarefaction was performed independently within each enrichment to allow comparison among samples from the same enrichment.

Following rarefaction, 16S rRNA gene copy number correction was applied using the rrnDB database (v5.10[12]). A custom script assigned mean copy numbers at the species, genus, or family level based on taxonomic matches. When no family-level value was available, a default copy number of 1 was used.

Relative abundances of each ASV were calculated from copy number-corrected counts, and absolute abundances were estimated by scaling relative abundances with sample-specific total cell counts from flow cytometry. Fold change for each ASV was calculated as:

$${Fold Change}_{i}= \frac{absolute abundance of {ASV}_{i} at t_{end}}{absolute abundance of {ASV}_{i} at t_{0}}$$

After fold change calculations, ASVs were clustered into OTUs to minimise the impact of suspected technical noise. Within each genus, ASVs were aligned to the most abundant sequence and clustered based on pairwise genetic distances, using a 0.0025 distance threshold to group sequences into OTU-like clusters. This threshold was chosen after examining mutation counts and phylogenetic tree structure to produce biologically sensible clusters.

Growth status for all isolation samples was determined from fold change values relative to the original inoculum. For both plate and droplet cultivation under autotrophic and heterotrophic conditions, presence/absence matrices were generated by comparing the fold for an ASV to a threshold of 2 (fold change>2 = growth, <2 no growth).

Growth status at the OTU level was assigned based on the most abundant ASV in each OTU, which served as the representative. The same principle was applied to determine growth status at the genus level. This approach was chosen because low-abundance ASVs were putatively attributable to technical artefacts, and focusing on the dominant ASV served to reduce their influence.

#### 1.1.2.2 Assessing isolation success

To estimate isolation performance for each cultivation method, ASVs present in all inoculum samples per enrichment above a threshold of five reads were identified as the set of potentially isolatable taxa. Growth success was defined as the proportion of these taxa that exhibited growth during cultivation. This approach provided a standardised proxy for comparing isolation outcomes between methods.

Because inoculation depth varied between methods, in Part 4 of this study, the inoculum data were normalised to ensure fair comparisons. This normalisation was done as follows: for each pairwise comparison, only ASVs with a ≥95% probability of being detected by the method with the lowest inoculation depth were considered. This detection probability was transformed into a relative abundance cut-off by:

$$P_{miss}={(1-p)}^{n}$$

with P_miss_, the chance of missing an ASV, p the minimum relative abundance of the ASV and n the sample size. For a sample size of 1000 and a desired minimum probability of 95% (or 5% chance of missing), this becomes:

$$0.05=\left( 1-p \right)^{1000}$$

Solving for p gives:

$$p=0.003$$

OTUs meeting this relative abundance threshold were used as the common reference set for assessing growth success in both methods (plate-based isolation and droplet-based isolation), ensuring evaluations were made on equal terms regardless of differences in sampling depth.

#### 1.1.2.3 Statistical data analysis of droplet and plate recovery

All data and statistical analyses were conducted in R (version 4.5.1)[13] using functions from base R with following libraries: tidyverse for data wrangling[14], ggplot2 for plotting[15], DECIPHER for multiple alignment and phylogenetic distance calculations[16]

To compare the isolation success of droplets and plates, we used a paired one-sided bootstrap test (resampling over Enrichment × Bioreplicate pairs). This test estimates the probability that the mean paired difference (droplet - plate) is less than or equal to zero by resampling the observed differences with replacement (B = 2000 iterations). We chose this approach over the paired Wilcoxon signed-rank test because diagnostic checks (histograms, QQ plots, and symmetry plots of the paired differences) indicated skewness and poor symmetry, violating Wilcoxon’s distributional assumptions.

#### 1.1.2.4 Phylogenetic data analysis

Phylogenetic analyses were performed in R using the packages DECIPHER and ggtree. All ASV sequences were aligned with the AlignSeqs function in DECIPHER. Pairwise sequence dissimilarities were then calculated using the DistanceMatrix function, applying uncorrected p-distances and ignoring terminal gaps. The resulting distance matrix was used to construct a Neighbor-Joining (NJ) tree, which was visualized using ggtree.

## 1.2 Supplementary results

Figure S 1 Headspace pressure of enrichments over time shows biological gas consumption. Enrichments were diluted twice weekly. Solid points and connecting lines show measured headspace pressure. Open circles mark replenishment to around 120 kPa for all enriched reactors at same time as tenfold dilution. Dashed line indicates atmospheric pressure.

Figure S 2. Scan of heterotrophic plates from three enrichment sources, shown in biological duplicates. Top to bottom: methylotrophic reactor enrichment, aerobic sludge enrichment, and hydrogen reactor enrichment.

Figure S 3 Scan of autotrophically grown plates from three enrichment sources, shown in biological duplicates. Top to bottom: methylotrophic reactor enrichment, aerobic sludge enrichment, and hydrogen reactor enrichment.

Figure S 4 Microscopy image of autotrophically grown culture from aerobic sludge enrichment. Grown bacteria in droplets are visible in the middle left and middle right. Scale bar: 30 µm.

Figure S 5 Microscopy image of heterotrophically grown aerobic sludge enrichment. Bacterial growth within droplets is visible in the top half and middle of the bottom section. Scale bar: 30 µm.

Figure S 6 Microscopy image of methylotrophic reactor enrichment under autotrophic conditions. Scale bar: 30 µm.

Figure S 7 Microscopy image of methylotrophic reactor enrichment under heterotrophic conditions. Scale bar: 30 µm.

Figure S 8 Microscopy image of hydrogen reactor enrichment under heterotrophic conditions. Scale bar: 30 µm.

Figure S 9 **SYBR Green staining showed clear DNA-containing biomass above the background signal of the blank controls, supporting growth in the cultivated samples.** Endpoint fluorescence distributions are shown for the different enrichments and cultivation conditions, with biological replicates overlaid.

Figure S 10 Boxplots of normalized fold changes (fold change/median) per group. The fold changes were normalized by dividing the fold change of each OTU by the median of their group (The groups consist of every unique combination of Bioreplicate, Enrichment, Method, Condition). a) A detailed view (0-40) of the boxplots, b) a full-range overview of the boxplots.

Table S 1 Number of amplicon sequence variants (ASVs) shared between biological duplicates of each enrichment inoculum. Only ASVs with more than 5 reads in rarefied data were included. Top 30 most numerous genera are shown.

| **Enrichment** | **Taxon** | **nIDs** |
| --- | --- | --- |
| Methyl | G_Paracoccus | 113 |
| Aero | G_Xanthobacter | 92 |
| H2reactor | G_Hydrogenophaga | 74 |
| H2reactor | G_Acinetobacter | 65 |
| H2reactor | G_Rhodobacter | 36 |
| Aero | G_Aquamicrobium | 26 |
| Aero | G_Proteiniclasticum | 18 |
| Aero | G_Bacteroides | 14 |
| Aero | G_Hydrogenophaga | 14 |
| Aero | G_Pseudomonas | 13 |
| Aero | G_Achromobacter | 12 |
| H2reactor | G_Pseudomonas | 12 |
| Aero | G_Paracoccus | 10 |
| Methyl | G_Pseudomonas | 9 |
| Aero | G_ Rhizobium | 8 |
| Methyl | G_Azospirillum | 8 |
| Methyl | G_Devosia | 8 |
| Methyl | G_Shewanella | 8 |
| Methyl | G_Acetoanaerobium | 6 |
| Aero | G_Acinetobacter | 5 |
| Aero | G_Lachnoclostridium | 5 |
| Aero | G_Macellibacteroides | 5 |
| Aero | G_Sphaerochaeta | 5 |
| Methyl | G_Shinella | 5 |
| Methyl | F_Rhodobacteraceae | 4 |
| Methyl | G_Hydrogenophaga | 4 |
| Methyl | G_Lachnoclostridium | 4 |
| Methyl | G_Methyloversatilis | 4 |
| Aero | F_Xanthobacteraceae | 3 |
| H2reactor | G_Pseudoxanthomonas | 3 |

Table S 2 **OTU-level cultivation outcomes for selected taxa across droplet- and plate-based cultivation.** For each OTU, the enrichment source and cultivation condition are shown together with whether growth was detected following droplet or plate cultivation. The table highlights OTUs that were not recovered by one or both cultivation methods. The table counts growth in at least one replicate as growth for that method.

| **OTU name** | **Enrichment and cultivation condition** | **Cultivated in droplets?** | **Cultivated on plates?** |
| --- | --- | --- | --- |
| G_Lacunisphaera_1_H | H2reactor, hetero | No | No |
| G_Hydrogenophaga_7_H | H2reactor, hetero | Yes | No |
| G_Hydrogenophaga_6_H | H2reactor, hetero | Yes | No |
| G_Ferrovibrio_1_M | Methyl, auto | Yes | No |
| G_Bosea_1_H | H2reactor, hetero | Yes | No |

Table S 3 **Observed cell yield per occupied droplet under low- and high-occupancy conditions.** Theoretical droplet occupancies were 3.9% and 86.5% for the low- and high-occupancy conditions, respectively. Observed cell numbers per occupied droplet are shown as mean ± SD. The final column shows the theoretical relative nutrient availability per occupied droplet under the assumption of complete cross-droplet redistribution of the growth-limiting nutrient.

| **Enrichment** | **Occupancy condition** | **Theoretical occupancy** | **Observed cells per occupied droplet (mean ± SD)** | **Theoretical nutrient availability per occupied droplet if fully leaky** |
| --- | --- | --- | --- | --- |
| Aero | Low occupancy | 3.9% | 50.0 ± 14.1 | 25.6× |
| Aero | High occupancy | 86.5% | 44.4 ± 15.7 | 1.16× |
| Methyl | Low occupancy | 3.9% | 36.7 ± 4.7 | 25.6× |
| Methyl | High occupancy | 86.5% | 55.5 ± 7.9 | 1.16× |

### 1.2.1 Origin of full-length 16S microdiversity.

Considering the restrictive enrichment conditions, the high 16S ASV diversity observed in several genera (Table S 1) was striking. Because there are limited reports in the literature assessing such diversity at full-length 16S resolution under these conditions, we sought to report this result, to determine whether it reflects true strain- or species-level variation or technical artefacts. We therefore carried out two complementary analyses: (i) we examined the prevalence of ASVs from dominant genera across all samples to determine whether the same ASVs reappeared consistently and (ii) we assessed the phylogenetic clustering of the shared ASVs within these genera to evaluate the differences exhibited by the ASVs.

Analysis of ASV prevalence in similar inoculum samples (end of enrichment, inoculum bioreplicate 1 and 2, and – for aerobic sludge enrichment and methylotrophic enrichment –bioreplicate 3 and 4) from the three dominant genera revealed consistently high numbers of unique ASVs per sample. In the aerobic sludge inoculum, *Xanthobacter* comprised approximately 90 ASVs per sample (100, 95, 92, 92 and 91, respectively). *Hydrogenophaga* in the hydrogen reactor samples showed comparable richness, with 75, 76 and 79 ASVs. *Paracoccus* in the methylotrophic reactor enrichment samples was the richest, with 121, 120, 118, 135 and 104 ASVs across the five samples (*Figure S 11*a). For all groups, the proportion of ASVs shared across samples decreased with each additional sample considered yet remained relatively high when all samples were included. *Xanthobacter* had 86.5 % shared ASVs across all samples, for *Hydrogenophaga* this number was 79.3 % and for *Paracoccus* it was the lowest with 67.3 % ASVs shared.

Figure S 11 (a) Shared, cumulative and unique amplicon sequence variants (ASVs) over ordered samples for three enrichment types: Aerobic sludge enrichment (Aero, green), hydrogen reactor enrichment (H_2_reactor, blue), and Methylotrophic enrichment (Methyl, red). Thick solid lines indicate the cumulative number of unique ASVs per genus; dashed lines indicate the fraction of ASVs shared across all samples in the order; points represent the number of ASVs present in each individual sample. (b–d) Phylogenetic trees of a representative fraction of the ASVs of the dominant genera within each enrichment – (b) Xanthobacter in Aero, (c) Hydrogenophaga from H_2_reactor, and (d) Paracoccus from Methyl – with branch tip sizes proportional to average relative abundance and coloured by the number of mutations relative to the reference sequence. The tree was constructed using Neighbour joining based on p-distances of aligned sequences. X-axis shows the amount of nucleotide substitutions per site. The most abundant ASV within one genus was taken as the reference sequence each time. For Xanthobacter, mutation profiles relative to the reference sequence are shown (e) f) g)), with black lines marking mutation positions and cyan lines showing the rolling mean mutation density to highlight clusters of mutations.

We performed phylogenetic analysis of the prevailing ASVs for the three dominant genera to assess the degree of sequence divergence. To do this, we compared each variant to the most abundant sequence, determined the phylogenetic distance of aligned sequences (uncorrected p-distances), performed clustering (neighbour joining) and counted the number of mutations per sequence compared to the dominant ASV. In the *Xanthobacter* dataset from aerobic sludge inoculum samples, the most abundant ASV was accompanied by a cluster of little-prevalent sequences differing by a single nucleotide. A second cluster, centred on the second most abundant ASV, comprised sequences with low abundance with either one or two nucleotide differences. In addition, three ASVs differed substantially from the dominant type, with 17, 29 and 38 mutations, respectively (Figure S 11 b). Similar patterns were observed for *Hydrogenophaga* and *Paracoccus* (Figure S 12- Figure S 14), where its most abundant ASV is accompanied by many single mutation ASVs of low abundance. In both genera, this pattern was also evident for lesser-dominant ASVs, visible on the upper branches of Figure S 11 c and Figure S 11 d. For *Hydrogenophaga*, five distinct clusters were identified, while *Paracoccus* displayed three clusters apart from the dominant branch.

To further characterise the nature of these variants, we profiled the distribution of mutations relative to the dominant ASV for *Xanthobacter*. In the single-mutation clusters, most mutations occurred only once, suggesting random variation (Figure S 11 g). However, in some cases, such as the upper cluster (Figure S 11 e), certain positions showed recurrent mutations, suggesting possible phylogenomic signal. The middle cluster showed many recurrent mutations, which, together with the phylogenetic distance led us to conclude true phylogenomic variability (Figure S 11 f).

Based on a mock community analysis, where similar ‘single-nucleotide difference’ clustering patterns were observed (see SI, Figure S 15-S 22), a 99.75% identity threshold was selected as it grouped the majority of error-derived variants with their corresponding reference sequences. Accordingly, ASVs were clustered into OTUs at 99.75% 16S identity, retaining major phylogenetic clusters while grouping artefactual single-nucleotide variants. However, as a trade-off some true single-nucleotide differences that were detected in the mock community were lost. These OTUs serve as a denoised unit for assessing cultivation success of microdiversity at a sub-species level.

Figure S 12 Distribution of mutations relative to the reference, with per-position counts (bars) and rolling mean smoothing (cyan line). All amplicon sequence variants (ASVs) from Xanthobacter in the aerobic sludge enrichment are compared to the most abundant ASV of Xanthobacter, with the yellow zone indicating the V3–V4 region

Figure S 13 Distribution of mutations relative to the reference, with per-position counts (bars) and rolling mean smoothing (cyan line). All amplicon sequence variants (ASVs) from the hydrogen reactor enrichment are compared to the most abundant ASV of Hydrogenophaga, with the yellow zone indicating the V3–V4 region

Figure S 14 Distribution of mutations relative to the reference, with per-position counts (bars) and rolling mean smoothing (cyan line). All amplicon sequence variants (ASVs) from the methylotrophic reactor enrichment are compared to the most abundant ASV of Paracoccus, with the yellow zone indicating the V3–V4 region

Figure S 15 Phylogenetic clustering of 16S rRNA gene sequences of Bacillus subtilis based on pairwise nucleotide distances. Data from mock community sequencing. Sequences were aligned and clustered using a distance threshold of 0.25% (99.75% identity). Each tip represents an individual ASV or reference sequence, with point size indicating sequence type (reference or sample-derived) and color representing the number of nucleotide substitutions relative to the reference sequence. Numbers indicate cluster assignments based on hierarchical clustering. The x-axis reflects nucleotide substitutions per site. Mutation counts exclude terminal gaps and are calculated relative to the reference sequence used as alignment anchor.

Figure S 16 Phylogenetic clustering of 16S rRNA gene sequences of Enterococcus faecalis based on pairwise nucleotide distances. Data from mock community sequencing. Sequences were aligned and clustered using a distance threshold of 0.25% (99.75% identity). Each tip represents an individual ASV or reference sequence, with point size indicating sequence type (reference or sample-derived) and colour representing the number of nucleotide substitutions relative to the reference sequence. **Numbers indicate cluster assignments based on hierarchical clustering**. The x-axis reflects nucleotide substitutions per site. Mutation counts exclude terminal gaps and are calculated relative to the reference sequence used as alignment anchor.

Figure S 17 Phylogenetic clustering of 16S rRNA gene sequences of Escherichia coli based on pairwise nucleotide distances. Data from mock community sequencing. Sequences were aligned and clustered using a distance threshold of 0.25% (99.75% identity). Each tip represents an individual ASV or reference sequence, with point size indicating sequence type (reference or sample-derived) and colour representing the number of nucleotide substitutions relative to the reference sequence. **Numbers indicate cluster assignments based on hierarchical clustering**. The x-axis reflects nucleotide substitutions per site. Mutation counts exclude terminal gaps and are calculated relative to the reference sequence used as alignment anchor.

Figure S 18 Phylogenetic clustering of 16S rRNA gene sequences of Lactobacillus fermentum based on pairwise nucleotide distances. Data from mock community sequencing. Sequences were aligned and clustered using a distance threshold of 0.25% (99.75% identity). Each tip represents an individual ASV or reference sequence, with point size indicating sequence type (reference or sample-derived) and colour representing the number of nucleotide substitutions relative to the reference sequence. **Numbers indicate cluster assignments based on hierarchical clustering**. The x-axis reflects nucleotide substitutions per site. Mutation counts exclude terminal gaps and are calculated relative to the reference sequence used as alignment anchor.

Figure S 19 Phylogenetic clustering of 16S rRNA gene sequences of Listeria monocytogenes based on pairwise nucleotide distances. Data from mock community sequencing. Sequences were aligned and clustered using a distance threshold of 0.25% (99.75% identity). Each tip represents an individual ASV or reference sequence, with point size indicating sequence type (reference or sample-derived) and colour representing the number of nucleotide substitutions relative to the reference sequence. **Numbers indicate cluster assignments based on hierarchical clustering**. The x-axis reflects nucleotide substitutions per site. Mutation counts exclude terminal gaps and are calculated relative to the reference sequence used as alignment anchor.

Figure S 20 Phylogenetic clustering of 16S rRNA gene sequences of Pseudomonas aeruginosa based on pairwise nucleotide distances. Data from mock community sequencing. Sequences were aligned and clustered using a distance threshold of 0.25% (99.75% identity). Each tip represents an individual ASV or reference sequence, with point size indicating sequence type (reference or sample-derived) and colour representing the number of nucleotide substitutions relative to the reference sequence. **Numbers indicate cluster assignments based on hierarchical clustering**. The x-axis reflects nucleotide substitutions per site. Mutation counts exclude terminal gaps and are calculated relative to the reference sequence used as alignment anchor.

Figure S 21 Phylogenetic clustering of 16S rRNA gene sequences of Salmonella enterica based on pairwise nucleotide distances. Data from mock community sequencing. Sequences were aligned and clustered using a distance threshold of 0.25% (99.75% identity). Each tip represents an individual ASV or reference sequence, with point size indicating sequence type (reference or sample-derived) and colour representing the number of nucleotide substitutions relative to the reference sequence. **Numbers indicate cluster assignments based on hierarchical clustering**. The x-axis reflects nucleotide substitutions per site. Mutation counts exclude terminal gaps and are calculated relative to the reference sequence used as alignment anchor.

Figure S 22 Phylogenetic clustering of 16S rRNA gene sequences of Staphylococcus aureus based on pairwise nucleotide distances. Data from mock community sequencing. Sequences were aligned and clustered using a distance threshold of 0.25% (99.75% identity). Each tip represents an individual ASV or reference sequence, with point size indicating sequence type (reference or Sequenced mock community) and colour representing the number of nucleotide substitutions relative to the reference sequence. **Numbers indicate cluster assignments based on hierarchical clustering**. The x-axis reflects nucleotide substitutions per site. Mutation counts exclude terminal gaps and are calculated relative to the reference sequence used as alignment anchor. Mock community used

## 1.3 Supplementary references

1.	Favere J, Waegenaar F, Jia M, Folens K, Verhoeven M, Balliu E *et al.* Production of biostable drinking water using a lab-scale biological trickling filter enriched with hydrogen-oxidizing bacteria. *NPJ Clean Water* 2024;**7**(1):1–21. https://doi.org/10.1038/s41545-024-00396-5.

2.	Heyse J, Props R, Kongnuan P, Schryver P De, Rombaut G, Defoirdt T *et al.* Rearing water microbiomes in white leg shrimp (Litopenaeus vannamei) larviculture assemble stochastically and are influenced by the microbiomes of live feed products. *Environ Microbiol* 2021;**23**(1):281–98. https://doi.org/10.1111/1462-2920.15310.

3.	Nauwynck W, Faust K, Boon N. Accessible, robust ultrahigh-throughput microbiome analysis via integration of uniform droplet-templated emulsification and FACS. *PLoS One* 2026;**21**(9):e0356887. https://doi.org/10.1371/JOURNAL.PONE.0356887.

4.	PacificBiosciences. GitHub - PacificBiosciences/pbbioconda: PacBio Secondary Analysis Tools on Bioconda. Contains list of PacBio packages available via conda. https://github.com/PacificBiosciences/pbbioconda (14 August 2025, date last accessed).

5.	PacificBiosciences/HiFi-16S-workflow: Nextflow pipeline to analyze PacBio HiFi full-length 16S data. https://github.com/PacificBiosciences/HiFi-16S-workflow (14 August 2025, date last accessed).

6.	Martin M. Cutadapt removes adapter sequences from high-throughput sequencing reads. *EMBnet J* 2011;**17**(1):10. https://doi.org/10.14806/ej.17.1.200.

7.	Callahan BJ, McMurdie PJ, Rosen MJ, Han AW, Johnson AJA, Holmes SP. DADA2: High-resolution sample inference from Illumina amplicon data. *Nat Methods* 2016;**13**(7):581–3. https://doi.org/10.1038/nmeth.3869.

8.	Quast C, Pruesse E, Yilmaz P, Gerken J, Schweer T, Yarza P *et al.* The SILVA ribosomal RNA gene database project: Improved data processing and web-based tools. *Nucleic Acids Res* 2013;**41**(D1):D590–6. https://doi.org/10.1093/nar/gks1219.

9.	Mahler L, Niehs SP, Martin K, Weber T, Scherlach K, Hertweck C *et al.* Highly parallelized droplet cultivation and prioritization of antibiotic producers from natural microbial communities. *Elife* 2021;**10**:e64774. https://doi.org/10.7554/eLife.64774.

10.	Scott GD, Kilgour DM. The density of random close packing of spheres. *J Phys D Appl Phys* 1969;**2**(6):863–6. https://doi.org/10.1088/0022-3727/2/6/311.

11.	Seo EY, Suzuki R, Takagi Y, Kindaichi T, Ohashi A, Kato S *et al.* High-density microdroplet cultivation reveals the essential role of microbial interactions in the growth of environmental microbes. *mBio* 2026;**17**(5):e0395325. https://doi.org/10.1128/mbio.03953-25.

12.	Stoddard SF, Smith BJ, Hein R, Roller BRK, Schmidt TM. rrnDB: Improved tools for interpreting rRNA gene abundance in bacteria and archaea and a new foundation for future development. *Nucleic Acids Res* 2015;**43**(D1):D593–8. https://doi.org/10.1093/nar/gku1201.

13.	Li J. The Exploration of the Approach to Data Preparation for Chinese Text Analysis Based on R Language. OALib. Vienna, Austra, 2021, 1–8. https://doi.org/10.4236/oalib.1107821.

14.	Wickham H, Averick M, Bryan J, Chang W, McGowan L, François R *et al.* Welcome to the Tidyverse. *J Open Source Softw* 2019;**4**(43):1686. https://doi.org/10.21105/joss.01686.

15.	Valero-Mora PM. *ggplot2: Elegant Graphics for Data Analysis* . New York: Springer-Verlag, 2010. https://doi.org/10.18637/jss.v035.b01.

16.	Wright ES. Using DECIPHER v2.0 to analyze big biological sequence data in R. *R Journal* 2016;**8**(1):352–9. https://doi.org/10.32614/rj-2016-025.
